# Aquaporin 1 elicits cell motility and coordinates vascular bed formation by downregulating thrombospondin type‐1 domain‐containing 7A in glioblastoma

**DOI:** 10.1002/cam4.3032

**Published:** 2020-04-06

**Authors:** Masahiro Oishi, Seiichi Munesue, Ai Harashima, Mitsutoshi Nakada, Yasuhiko Yamamoto, Yasuhiko Hayashi

**Affiliations:** ^1^ Department of Neurosurgery Kanazawa University Graduate School of Medical Sciences Kanazawa Japan; ^2^ Department of Biochemistry and Molecular Vascular Biology Kanazawa University Graduate School of Medical Sciences Kanazawa Japan; ^3^ Department of Neurosurgery Kanazawa Medical University Uchinada Japan

**Keywords:** aquaporin‐1, glioblastoma, THSD7A, tube formation, vascular bed

## Abstract

**Background:**

Aquaporin (AQP) 1 expression has been linked with tumor malignancy but its role in glioblastoma (GBM), a lethal glioma, remains to be clarified.

**Methods:**

AQP1 expression was examined in 33 human GBM specimens by immunohistochemistry. GBM cells (U251 and U87) that stably express AQP1 were established and used for cellular proliferation, migration, invasion, and vascular tube formation assays. The GeneChip assay was used to identify differentially expressed genes in AQP1‐expressing cells.

**Results:**

AQP1 was expressed only in tumor cells. AQP1 dose‐dependently accelerated cell migration and invasion, but not proliferation, in GBM cell lines. AQP1 also upregulated cathepsin B, focal adhesion kinase and activities of matrix metalloproteinase 9. AQP1 in GBM cells induced wall thickness of ECV304, vascular endothelial cells, in a contact‐dependent manner. Downregulation of thrombospondin type 1 domain containing 7A (THSD7A) was identified in AQP1‐expressing GBM cells in vitro, and was negatively correlated with AQP1 expression in human GBM specimens.

**Conclusion:**

AQP1 is involved in tumor malignancy by facilitating the migration and invasion of GBM cells, and promoting the formation of vascular beds that are characteristic of GBM by downregulating THSD7A.

## Graphical abstract

We examined whether AQP1 could enhance the malignant properties of GBM in vitro, including cell proliferation, migration, invasion, and blood vessel‐like tube formation. AQP1 is involved in tumor malignancy by facilitating the migration and invasion of GBM cells, and promoting the formation of vascular beds that are characteristic of GBM by downregulating THSD7A.

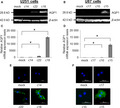

## 1 INTRODUCTION

Gliomas are the most common primary brain tumors. Astrocytoma, a type of glioma, can be categorized into grades I‐IV, from the lowest to the highest degree of malignancy, according to the World Health Organization Classification of Tumors. Astrocytoma grade Ⅳ, which is known as glioblastoma (GBM), is the most malignant brain tumor. The median survival for patients with GBM is still only approximately 16 months postdiagnosis, even when multidisciplinary treatment is provided to the patients.^1^ GBM is characterized by its high capacity for proliferation, invasion, and extensive vascularization; however, the precise mechanisms underlying these aggressive tendencies have not been fully elucidated.

Aquaporins (AQPs) are small channel‐forming transmembrane proteins that facilitate rapid transport of water and small solutes across biological membranes.^2^ Thirteen AQPs have been identified in humans.^3^ AQP1 was the first AQP discovered in mammals.^4^ AQP1 expression has been reported in the choroid plexus, kidney, corneal endothelium, and vascular endothelial cells.^5^, ^6^ In addition, AQP1 is overexpressed in various human cancers, including biliary duct, bladder, breast, cervix, lung, prostate, nasopharynx, and brain cancers.^7^ Previous studies have shown that the expression of AQP1 is positively associated with invasion, angiogenesis, cell migration, and the formation of edema in malignant tumors,^7^, ^8^, ^9^, ^10^, ^11^ and high‐grade astrocytomas express higher levels of AQP1 than low‐grade astrocytomas.^12^ Upregulation of AQP1 occurs predominantly in the perivascular space that is distant from the necrotic tumor core and has high tumor infiltration.^8^ In addition, tumor microvessel proliferation is impaired in AQP1‐deficient mice.^11^ This evidence suggests that AQP1 upregulation causes an increase in blood vessel formation.

Thrombospondin type‐1 domain‐containing 7A (THSD7A) is a membrane‐associated N‐glycoprotein. It has been linked to the pathogenesis of membranous nephropathy (MN),^13^, ^14^ obesity,^15^ and angiogenesis.^16^, ^17^, ^18^ Membrane‐associated THSD7A can associate with the αvβ3 integrin‐paxillin focal adhesion complex and can inhibit human umbilical vein endothelial cell (HUVEC) migration and tube formation.^17^ On the other hand, soluble THSD7A, a secreted form of the membrane‐associated THSD7A, can promote endothelial cell migration, tube formation, and sprouting during angiogenesis in zebrafish and in HUVECs.^16^, ^18^ However, the involvement of THSD7A in GBM malignancies, particularly in angiogenesis, is completely unknown.

In this study, we examined whether AQP1 could enhance the malignant properties of GBM in vitro, including cell proliferation, migration, invasion, and blood vessel‐like tube formation. Moreover, human GBM specimens were subjected to immunohistological analyses to validate the in vitro results. The results of this study may enhance our understanding of the mechanisms underlying the involvement of AQP1 in GBM malignancy.

## 2 MATERIALS AND METHODS

### 2.1 Human GBM specimens

All the clinical GBM tissue samples (33 cases) were obtained from Kanazawa University Hospital (Kanazawa, Japan) and, according to criteria from the World Health Organization (WHO), classified into four grades with increasing malignancy: Grade I pilocytic astrocytoma; grade II astrocytoma; grade III anaplastic astrocytoma; and grade IV GBM, the most malignant brain tumor (Table 1). The present study was approved by the Institutional Review Boards of the Kanazawa University Hospital (#2014‐033[1637]), and all participants provided written informed consent.

**TABLE 1:** Clinical characteristics of thirty‐three patients with glioblastoma

Case	Age	Sex	Location	AQP1	THSD7A
1	77	F	Rt‐frontal	4	0
2	63	M	Lt‐frontal~insula	3	1
3	75	M	Lt‐parietal~occipital	4	0
4	41	M	Lt‐parietal	3	0
5	56	M	Rt‐temporal	2	0
6	76	F	Lt‐frontal	3	0
7	54	M	Lt‐temporal	4	1
8	42	M	Lt‐temporal	3	0
9	77	M	Lt‐frontal	2	0
10	67	F	Lt‐parietal	4	1
11	67	F	Rt‐frontal	2	1
12	78	M	Lt‐frontal	4	1
13	68	F	Lt‐parietal	2	1
14	53	M	Lt‐frontal	3	0
15	70	M	Lt‐temporal	4	2
16	79	F	Rt‐parietal	4	2
17	83	M	Lt‐occipital	4	0
18	76	M	Lt‐frontal	4	0
19	72	M	Rt‐frontal	4	1
20	39	M	multicentric	4	0
21	71	M	multicentric	3	0
22	80	F	Lt‐frontal	3	1
23	70	M	Rt‐temporal	3	1
24	68	M	Rt‐frontal	4	0
25	76	F	Rt‐frontal	4	0
26	77	M	Lt‐frontal	4	0
27	56	F	Lt‐frontal	4	0
28	48	F	Rt‐frontal	3	0
29	66	M	Rt‐temporal	3	1
30	35	F	Rt‐frontal	3	0
31	73	M	Lt‐temporal	3	2
32	55	F	Lt‐frontal	3	1
33	74	F	Lt‐frontal~insula	3	0

Note

Expression grading: 0 (none), 1 (<5%), 2 (5%‐50%), 3 (50%‐75%), and 4 (>75%).

Abbreviations: F, female; Lt, left; M, male; Rt, Right.

### 2.2 Immunohistochemistry

Immunohistochemistry (IHC) was performed as previously described.^19^ Thirty‐three GBM specimens were fixed in formalin and embedded in paraffin blocks. The tissue blocks were sectioned (4‐µm thick) onto slides and then deparaffinized in xylene, followed by dehydration in graded alcohol. The samples were then rinsed in water for 15 minutes. The slides were microwaved for 15 minutes in target retrieval solution (pH 6.0; Dako). Internal peroxidases were blocked by incubation in 0.3% H_2_O_2_ in methanol for 30 minutes. Nonspecific staining was blocked by incubation with the blocking solution consisting of Tris‐buffered saline (TBS) with 0.1% Tween 20 (TBS‐T) and 5% skimmed milk for 30 minutes. Sections were immunostained using the Envision^+^ Kit (Dako) according to manufacturer's instructions. The primary antibodies used were as follows: mouse monoclonal anti‐human CD34 (M7165, Dako; 1:400), rabbit polyclonal anti‐AQP1 (H‐55) (sc‐20810, Santa Cruz Biotechnology, Inc; 1:2000), and rabbit polyclonal anti‐THSD7A (HPA000923, Atlas Antibodies; 1:1000). The sections were exposed to diaminobenzidine peroxidase substrate (Funakoshi) and counterstained with Mayer's hematoxylin. Hematoxylin and eosin (HE) staining was performed as usual. The slides were scanned by a BZ‐X710 microscope (Keyence). Assessment of the sections was performed in a blinded fashion by two independent investigators.

### 2.3 Cell culture

Human glioma cell lines, U251 and U87 cells, were obtained from the American Type Culture Collection (ATCC^®^) in 2009. These cell lines were characterized in the resource institute by short tandem repeat profile analysis. Authentication of the cell lines was unnecessary because the cells were expanded by culturing them for less than two passages before storage at −80°C. Low‐passage cells were used for the experiments within 6 months after resuscitation. They were maintained in Dulbecco's Modified Eagle Medium (DMEM) supplemented with 10% fetal bovine serum (FBS) at 37°C with 5% CO_2_. All cells were mycoplasma‐free.

The HUVEC‐derived cell line, ECV304, was kindly and directly provided by Dr Takahashi of The National Defense Medical College of Japan where ECV304 was established.^20^ It was maintained in Medium 199 supplemented with 10% FBS at 37°C with 5% CO_2_.

### 2.4 Immunocytochemistry

The U251 and U87 cells were seeded in the 4‐well Lab‐Tek Ⅱ Chamber Slide system (Merck KGaA) and were fixed with 4% paraformaldehyde. They were incubated in 5% skimmed milk in TBS‐T at room temperature (RT) for 30 minutes to block nonspecific immunoreactions. Next, the sections were incubated overnight at 4°C with rabbit polyclonal anti‐AQP1 (H‐55) (sc‐20810, Santa Cruz Biotechnology, Inc; 1:500) and then with anti‐rabbit IgG secondary antibodies conjugated with Alexa Fluor‐488 (Thermo Fisher Scientific; 1:2000) at RT for 30 minutes. Nuclei were stained with 4′, 6‐diamidino‐2‐phenylindole (DAPI, Santa Cruz Biotechnology). All images were captured using a BZ‐X710 microscope.

### 2.5 Construction and transfection of expression vectors, and establishment of AQP1‐expressing cells

Human *AQP1* cDNA was amplified by polymerase chain reaction (PCR) using the following primers: 5′‐GAGAATTCTCAGGCCAAGCCCCCTGCCA‐3′ (nt 89‐108 of NM_198098), and 5′‐GAGTCGACACGTGGATGCCCGGGCCAGA‐3′ (nt 927‐946 of NM_198098), containing EcoRI and SalI sites (indicated by underlines) respectively. The PCR products were extracted from 0.8% low‐melting point agarose gel and digested with EcoRI and SalI. The PCR products were inserted into the pCI‐neo Mammalian Expression vector (Promega) at the EcoRI and SalI cloning sites to construct the pCI‐neo‐AQP1 expression vector. All ligation products were transformed into One Shot^®^ TOP10 chemically competent *E. coli* (Invitrogen). Recombinant plasmid DNAs were purified with a plasmid isolation kit (Qiagen). The constructs were confirmed by DNA sequencing.

For transfection, the U251 and U87 cells were seeded into 6‐well culture plates and grown until 80% confluence. The pCI‐neo‐AQP1 construct and the control (empty pCI‐neo) were stably transfected using the Lipofectamine 3000 reagent (Invitrogen) according to the manufacturer's instructions. G418 (500 μg/mL) was used to select stably transfected cells. Single‐cell clones were isolated using the limiting dilution method. The transfection efficiency was determined by qualitative real‐time (qRT)‐PCR and western blotting. Ten positive clones and three control clones were picked from each cell line after 20 days of selection. Next, three positive clones and a control clone were chosen from each cell line for further experiments. Stably transfected clones were subsequently cultured and amplified in culture medium supplemented with 500 μg/mL G418.

### 2.6 Western blotting

Western blotting analysis was carried out as previously described.^21^ Briefly, cell lysates containing 30 μg protein were boiled in Laemmli buffer and resolved by sodium dodecyl sulfate‐polyacrylamide gel electrophoresis (SDS‐PAGE) using a 12.5% gel and then transferred onto a polyvinylidene fluoride membrane (Millipore Corp.). The membranes were incubated with the primary antibodies rabbit polyclonal anti‐AQP1 (H‐55) (sc‐20810, Santa Cruz Biotechnology, Inc; 1:2000), polyclonal anti‐cathepsin B (Bio Vision; 1:500), and polyclonal FAK (Cell Signaling Technology, Inc; 1:500) with IRDye 680RD anti‐rabbit IgG as the secondary antibody. The immunoreactive bands were visualized using the Odyssey Infrared Imaging system (LI‐COR Biotechnology).

### 2.7 Quantitative real‐time polymerase chain reaction (qRT‐PCR)

Levels of mRNA were measured by qRT‐PCR. Total RNA was isolated from U251 and U87 cells with the RNeasy Plus Mini Kit (Qiagen), and complementary DNA was synthesized using the oligo(dT) primer of the AffinityScript QPCR cDNA Synthesis Kit (Agilent Technologies) according to the manufacturers' protocols. SYBR green qRT‐PCR was performed with specific DNA primers. Amplification and real‐time fluorescence detection were performed using the Mx3005P Real‐Time QPCR system (Stratagene Products Division, Agilent Technologies) with the following protocol: an activation step (95°C, 60 seconds), followed by 40 cycles of denaturation (95°C, 30 seconds), and annealing (55°C, 30 seconds). The mRNA levels of the genes were normalized against that of the TATA‐binding protein (TBP).

### 2.8 Cell proliferation assay

The alamarBlue assay (Biosource) was performed according to the manufacturer's protocol. Briefly, U251 cells (3 × 10^3^ cells/well) or U87 cells (7 × 10^3^ cells/well) in 100 μL of culture medium supplemented with 0.1% FBS were seeded in 96‐well plates. The cells were incubated for 4 hours at 37°C, and 10 μL alamarBlue (10% of total volume) was added to the cells. The plate was read at 570 and 610 nm with a standard spectrophotometer 4, 12, 24, 48, and 72 hours after the addition of the dye.

### 2.9 Cell migration assay

U251 and U87 cells were seeded at a density of 2 × 10^5^ and 1 × 10^6^ cells/well, respectively, and grown to 100% confluence in 24‐well plates. The cell cultures were scratched with a 200‐μL pipette tip to create a cell‐free zone (wound). The cells were washed twice with serum‐free DMEM and then further incubated in DMEM with 0.1% FBS. Percent migration (the area of the wound at 24 h/the area of the wound at 0 h) was assessed 24 hours after denudation using Image‐Pro Premier software (Nippon Roper).

### 2.10 Cell invasion assay

The invasive capacity of tumor cells was examined using 24‐well transwell plates (8‐µm pore size; Corning Incorporated) pre‐coated with Matrigel (BD Biosciences). The U251 and U87 cells (5 × 10^4^ cells/well in 500 µL serum‐free medium) were seeded in the upper chambers of wells, while 500 µL medium containing 1% FBS was placed in the lower chambers. After incubation at 37°C for 24 hours, noninvading cells on the upper side of the membrane were removed with a cotton swab, and the invading cells were fixed with methanol and stained with 0.1% crystal violet. The invading cells on the filter were counted from five randomly selected high‐power microscopic fields.

### 2.11 Matrix metalloproteinase activity assay

Matrix metalloproteinase‐9 (MMP‐9) activity was measured using a MMP‐9 activity assay kit (QuickZyme Biosciences) according to the manual. U251 and U87 cells were seeded at a density of 1 × 10^6^ and 3 × 10^6^ cells/well, respectively, and grown to 100% confluence in 6‐well plates. The cells were washed three times with serum‐free DMEM and then further incubated in serum‐free DMEM for 24 hours. The cultured media were collected for the assay and these cell number were counted using TC20™ automated cell counter (Bio‐Rad Laboratories, Inc). MMP‐9 activities were normalized to individual cell numbers.

### 2.12 Assay for cord‐like structure formation by ECV304 cells (in vitro angiogenesis)

We examined the in vitro formation of capillary‐like tube structures by ECV304 cells, human endothelial cells, seeded on Matrigel (Corning) by two methods. In the first method, ECV304 cells (8 × 10^4^ cells/well) were co‐cultured with the same number of U251 or U87 in 24‐well plates (Figure 4A) in Medium 199 containing 0.1% FBS at 37°C with 5% CO_2_. In the second method, 6 × 10^4^ ECV304 cells were seeded with 6 × 10^5^ U251 cells or 1 × 10^6^ U87 cells in a NICO‐1 (Ginrei Lab) Interactive Co‐Culture Plate (ICCP). NICO‐1 is a new ICCP in which the vessels are connected horizontally to allow sharing of the culture medium (Figure 5A). Using this method, the endothelial cells and GBM cells do not interact directly; however, they share the same culture medium, which is conditioned by both cell types.

Tube formation ability was measured after 24 hours using a BZ‐X710 microscope. Total tube lengths, total covered area, and theoretical average tube width were quantified with Image‐Pro Premier software (Nippon Roper). ECV304 cells were stained with PKH26 (red), according to the protocol suggested by the manufacturer (Sigma). The total tube lengths were determined with a self‐made length‐calculating using Image‐Pro Premier software. The total covered area were calculated automatically using Image‐Pro Premier software. The theoretical average tube width was calculated by dividing the covered area by the total tube length of the image.

### 2.13 GeneChip microarray profiling and associated bioinformatics analysis

Total RNA was extracted from U251‐c18 cells and the U251 mock‐transfected control cells using TRIzol (Invitrogen), and purified using the RNeasy Micro Kit and the RNase‐Free DNase Set (Qiagen GmBH). Comparative gene expression analysis was performed using the Affymetrix GeneChip Human Genome U133 Plus 2.0 Array (Affymetrix). Array washing, scanning, and probe quantification protocols were carried out according to the manufacturer's instructions using Affymetrix GeneChip Operating Software (GCOS) (http://www.affymetrix.com). For each array, the GCOS output was imported as CEL files into the Partek Genomics Suite software (Partek Inc). The gene expression data were quantified with the Robust Multichip Averaging algorithm and were normalized and corrected for multiple testing with the Benjamini‐Hochberg method for detection of differentially expressed genes. A gene was designated as differentially expressed if the change in the signal log ratio value was ≥2‐ or ≤0.5‐fold. qRT‐PCR of the candidate genes was used to confirm the results. All qRT‐PCR experiments were performed in triplicate.

### 2.14 Statistics

Statistical significance was determined using Student's *t* test and Mann‐Whitney *U* test for comparison of two groups when appropriate. All analyses were performed using JMP Statistical Discovery Software version 14.0 (SAS Institute Inc). The statistical significance level was set to *P* < .05.

## 3 RESULTS

### 3.1 Tumor cells in human GBM specimens express AQP1

**FIGURE 1:**
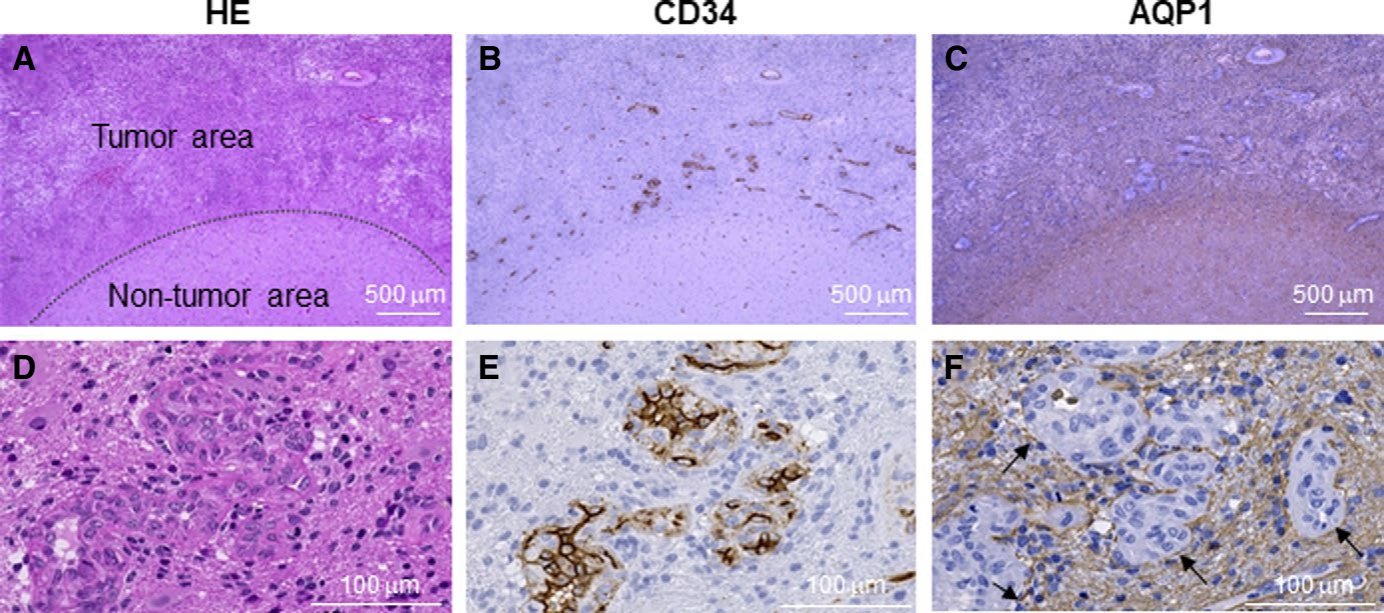
Tumor cells in human glioblastoma (GBM) specimens express AQP1 by immunohistochemistry. Representative images of GBM specimens from 33 patients analyzed by (A, D) hematoxylin‐eosin (HE) staining, (B, E) CD34 staining (to identify endothelial cells), and (C, F) AQP1 staining. For A‐C, scale bars = 500 µm; For D‐F, scale bars = 100 µm. Based on the proportion of AQP1‐expressing GBM cells, the cases were classified into different grades: 0 (none), 1 (<5%), 2 (5%‐50%), 3 (50%‐75%), and 4 (>75%)

To determine the expression and localization of AQP1 in GBM, we evaluated sections of GBM specimens from 33 patients by immunohistochemistry (Figure 1; Table 1). We probed for AQP1 and CD34 (to identify endothelial cells) and stained with hematoxylin and eosin (HE) (Figure 1A,D) to identify tumor and nontumor areas of the specimens. Endothelial cells in the tumor region did not express AQP1 (Figure 1B,E); however, AQP1 was highly expressed by the tumor cells in the tumor periphery, especially those in the perivascular zone (Figure 1C,F [arrows]). Glomeruloid neovascularization and several branching vessels were found in the tumor region near infiltration areas. We assigned a grade based on the proportion of tumor cells expressing AQP1 in each specimen: 0 (none), 1 (<5%), 2 (5%‐50%), 3 (50%‐75%), and 4 (>75%). In all the specimens at least 5%‐50% of the GBM cells expressed AQP1, and 50% to >75% of tumor cells expressing AQP1 (> grade 3) are found to be the majority of the specimens (29/33, approximately 88%) (Table 1). These data confirm that tumor cells in human GBM specimens express high levels of AQP1.

### 3.2 Creation and confirmation of U251 and U87 GBM clones that stably express exogenous AQP1

**FIGURE 2:**
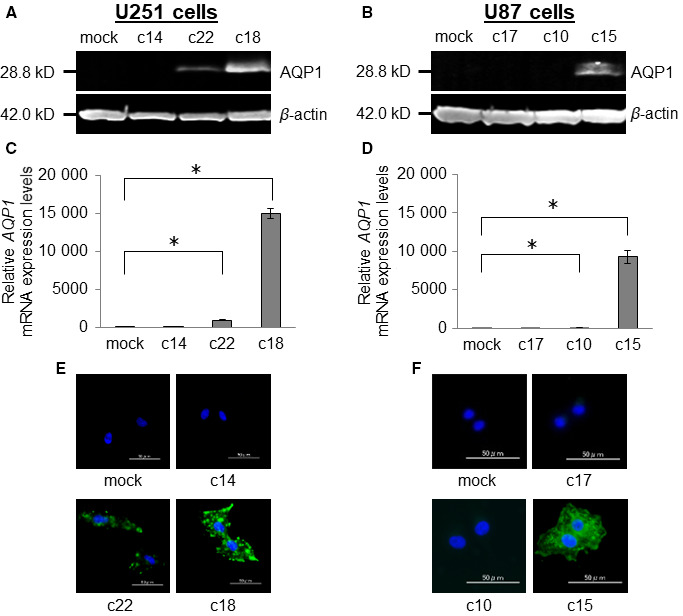
Creation and confirmation of U251 and U87 clones that stably express aquaporin 1 (AQP1). Expression of AQP1 in representative U251 and U87 clones transfected with pCI‐neo‐AQP1 and pCI‐neo (mock) control by (A, B) western blotting and by (C, D) qRT‐PCR (*P < .05). E and F, AQP1 expression in U251 and U87 clones by immunofluorescence microscopy; (blue: DAPI [nuclei], green: AQP1); scale bars = 50 µm

To explore the role of AQP1 in GBM in vitro, we established GBM cell lines that stably express human AQP1 at different levels. Human glioma cell lines U251 and U87 cells were transfected with either a human AQP1 expression vector or an empty vector (mock) as the control. Endogenous expression of AQP1 was undetectable in the mock controls of both U251 and U87 cells (Figure 2A,B respectively). After G418 selection (per manufacturer's instructions), we screened clones for AQP1 expression by western blot analysis (Figure 2A,B) and for *AQP1* mRNA levels by qRT‐PCR (Figure 2C,D). For each GBM cell line we selected three clones that displayed varying AQP1 expression (low, intermediate, and high): c14, c22, and c18 for the U251 cells; and c17, c10, and c15 for the U87 cells (Figure 2A‐D). To confirm these data, we evaluated AQP1 expression in the cells by immunofluorescence microscopy. Consistent with the qRT‐PCR and western blotting results, we found that the U251‐c18 and U87‐c15 clones had the highest AQP1 expression levels (Figure 2E,F respectively).

### 3.3 AQP1 enhances migration and invasion, but does not enhance the growth of U251 and U87 cells in vitro

**FIGURE 3:**
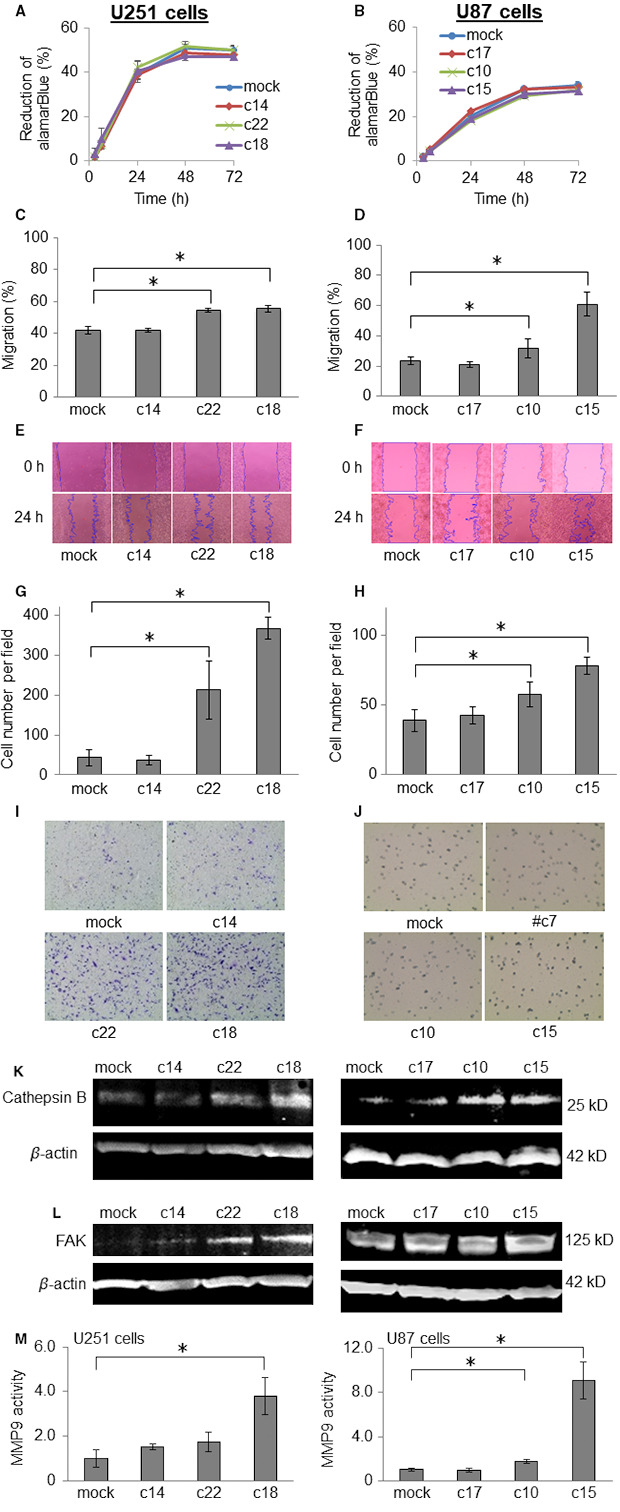
Aquaporin 1 (AQP1) enhances the migration and invasion of U251 and U87 cells in vitro, but does not enhance their growth. The comparative growth (A, B), migration (C‐F), and invasion (G‐J) of U251 and U87 cells engineered to exogenously express AQP1, and their mock‐transfected controls. A and B, The effect of AQP1 on the in vitro proliferation of U251 cells (A) and U87 cells (B), using cellular metabolism as a surrogate marker for growth. C‐F, The migration of U251 and U87 cells engineered to exogenously express AQP1, and their mock‐transfected controls, by wound‐healing assay. Percent migration ["Migration (%)"] was calculated as the area of the wound at 24 h/the area of the wound at 0 h. C and D, Data represent the mean ± SD of n = 6; *P < .05 for AQP1‐transfected cells vs mock‐transfected cells, Student's *t* test. E and F, Representative images of wound‐healing assay captured 0 and 24 h postwounding. The violet lines highlight the migrational front (edges of the wound). G‐J, Invasion from the upper to the lower chambers of Matrigel‐coated transwell plates by U251 and U87 cells engineered to exogenously express AQP1, and their mock‐transfected controls. G and H, The number of cells per visual field on the ventral surface of the transwell Matrigel‐coated filter 24 h after having been seeded in the upper chamber. Data represent the mean number of U251 (G) and U87 (H) cells per field ± SD of n = 6; *P < .05 for AQP1‐expressing cells vs mock‐transfected controls). I and J, Representative images of stable (I) U251 and (J) U87 transfectants that migrated to the ventral surface of transwell Matrigel‐coated filters, 24 h after having been seeded in the upper chamber. K and L, Western blot analysis of cathepsin B and focal adhesion kinase (FAK) levels in AQP1‐expressing U251 and U87 cells and the mock‐transfection control. M, Matrix metalloproteinase‐9 (MMP‐9) activities in U251 and U87 clones. Data represent the mean ± SD, n = 3; *P < .05 for AQP1‐expressing GBM clones vs their mock‐transfection controls

Compared with lower‐grade astrocytomas, GBMs express higher levels of AQP1, and are typified by enhanced proliferation, migration, and invasion; therefore, we evaluated these characteristics in vitro using the U251 and U87 clones that express exogenous levels of AQP1 and in their mock‐transfected controls. We observed no significant difference in proliferation between the AQP1‐expressing U251 and U87 clones and their controls (Figure 3A,B, respectively). To assess migration, we used a wound‐healing assay. Confluent monolayers of AQP1‐expressing U251 and U87 cells and control cells were scratched with a pipette tip to create a cell‐free zone/wound (0 hours). After 24 hours, we evaluated the percent migration of cells into the cell‐free zone. We found that AQP1 expression dose‐dependently and significantly increased the migration of both cell lines (Figure 3C‐F). To evaluate invasion in vitro, we assessed the migration of cells (from the upper to the lower chambers of transwell plates) through a filter coated with Matrigel, a reconstituted extracellular matrix used to mimic basement membrane (Figure 3G‐J). Compared with their respective mock controls, there were significantly more U251‐c22, U25‐c18, U87‐c10, and U87‐c15 AQP1‐expressing cells on the ventral surface of the transwell filters (Figure 3G‐J). These findings demonstrate that AQP1 promotes the invasion of U251 and U87 cells through Matrigel, and suggest that AQP1 may enhance the ability of GBM cells to invade surround tissues in vivo.

### 3.4 AQP1‐expressing GBM cells showed higher expression of cathepsin B and focal adhesion kinase and higher activities of MMP‐9

To characterize the potential mechanisms by which AQP1 enhances the migration and invasion of GBM cells in vitro, we evaluated the expression of cathepsin B and focal adhesion kinase (FAK), proteins that regulate cell migration and invasion, in the AQP1‐expressing U251 and U87 cells and control cells by western blot analysis.^22^, ^23^ Compared to control cells, the AQP1‐expressing U251 and U87 clones had higher levels of FAK and cathepsin B (Figure 3K.L). MMP‐9 activities were also significantly enhanced in AQP1‐expressing U251 cells as well as in AQP1‐expressing U87 cells (Figure 3M).

### 3.5 Direct co‐culturing with AQP1‐expressing GBM cells enhances the thickness and area covered, but does not affect the length of ECV304 cord‐like structures in vitro

**FIGURE 4:**
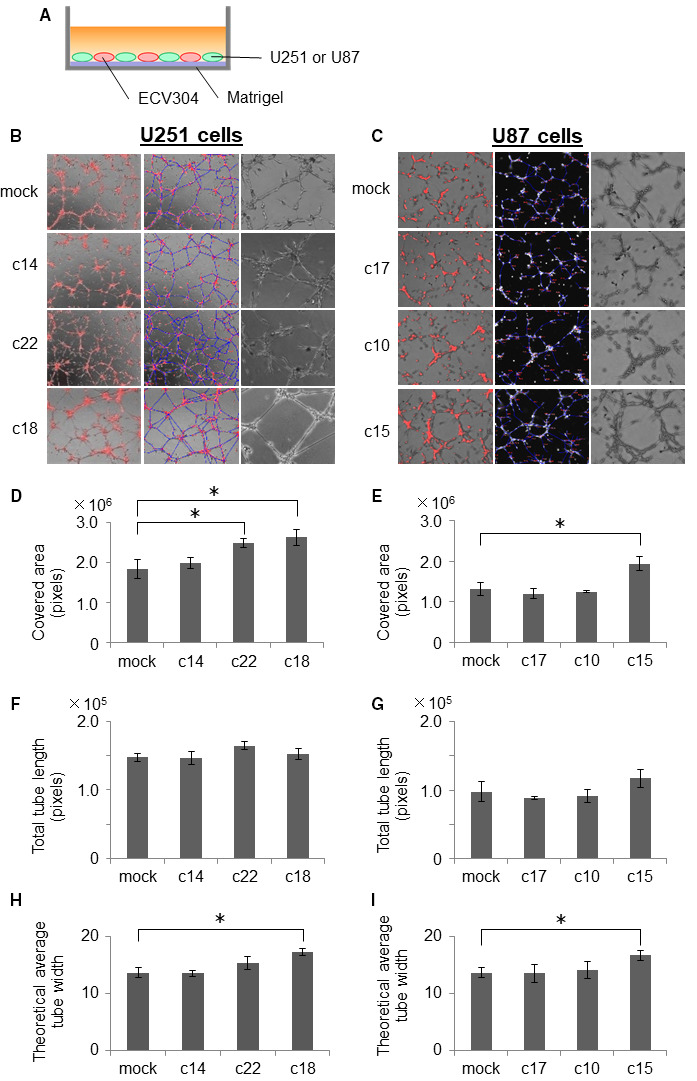
Coculturing endothelial cells with aquaporin 1 (AQP1)‐expressing glioblastoma (GBM) cells enhances the thickness and area covered but does not affect the length of ECV304 cord‐like structures in vitro*.* A, Schematic of tube formation assay of ECV304 human endothelial cells cocultured on Matrigel with U251 or U87 cells engineered to exogenously express AQP1, and their mock‐transfected controls. B and C, Representative photomicrographs of the tube formation of ECV304 cells 24 h after their coculture on Matrigel with U251 and U87 cell lines engineered to express AQP1, and their mock‐transfected controls; red indicates ECV304 cells labeled with PKH26. The blue line is the measurement line that calculates the tube length. Quantification of covered area (D, E), total tube length (F, G), and theoretical average tube width (H, I) of ECV304 cells from experiment described in A‐C. For D‐I, data represent the mean ± SD, n = 6; *P < .05 comparing ECV304 cells cocultured with AQP1‐expressing U251 or U87 cells vs their mock‐transfected controls

**FIGURE 5:**
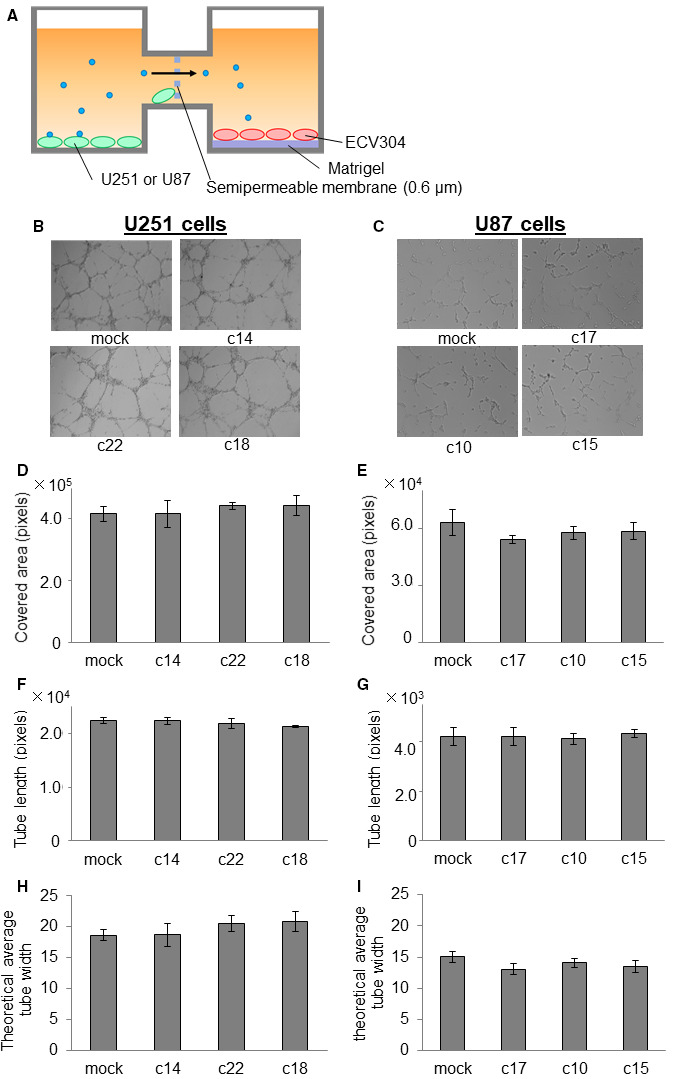
Direct cell‐to‐cell contact is required for aquaporin 1 (AQP1)‐expressing glioblastoma (GBM) cells to affect the width and area covered by endothelial cells in vitro. A, Schematic of tube formation assay in which ECV304 human endothelial cells and GBM cells engineered to exogenously express AQP1 (or not) are seeded in different chambers of a NICO‐1 co‐culture system, separated by a semipermeable membrane. Using this system, there is no direct contact between the cell types, but they share the same culture media. B and C, Representative photomicrographs of ECV304 cells after growing 24 h in media conditioned by GBM clones. Quantification of covered area (D, E), total tube length (F, G), and theoretical average tube width (H, I) of ECV304 cells from experiment described in A‐C. For D‐I, data represent the mean ± SD, n = 6

To assess if AQP1 expressed by GBM cells may affect the angiogenic potential of endothelial cells, we employed two in vitro co‐culture systems. One was a direct mixture of human endothelial cells (ECV304 cells) with U251 or U87 cells on Matrigel (Figure 4A), and the other was an indirect, interactive form of coculture using the NICO‐1 system, in which ECV304 cells and GBM cells were seeded in different culture chambers, but shared the same culture medium (Figure 5A). Using the direct cell mixture system, we labeled ECV304 cells with PKH26 (shown in red) to distinguish them from GBM cells and to evaluate the formation of cord‐like structures (Figure 4B,C).We found that ECV304 cells cocultured with the U251‐c18 and U87‐c15 cells, GBM clones that express the highest levels of AQP1, formed significantly thicker cord‐like structures compared to ECV304 cells incubated with the respective GBM mock‐infected controls (Figure 4B,C). Quantitative analyses revealed that incubating ECV304 cells with AQP1‐expressing U251 and U87 cells significantly increased the indices of covered area (Figure 4D,E) and theoretical average tube width (Figure 4H,I), but did not significantly affect the total tube length of ECV304 cells (Figure 4F,G). These findings suggest that AQP1 expression by GBM cells may enhance endothelial wall thickness and induce the formation of vascular beds.

### 3.6 The effect of AQP1‐expressing GBM cells on the width and area covered by ECV304 endothelial cells requires direct contact between the cell types in vitro

To examine whether a direct interaction between GBM and ECV304 cells was needed for endothelial cell tube formation, we used an indirect, interactive coculture system. In this system, ECV304 cells and GBM cells were seeded in different but connected plates/chambers, separated by a semipermeable membrane. Using this method, the endothelial cells and GBM cells did not interact directly; however, they shared the same culture medium, which was conditioned by both cell types (Figure 5A). Twenty‐four hours postseeding, we captured images of the ECV304 cells. Using these images, we quantified average ECV304 tube length, thickness, and area covered by the endothelial cells. We found no significant difference in these parameters for ECV304 cells cultured in media conditioned by AQP1‐expressing U251 and U87 cells compared with their respective mock‐infection controls (Figure 5B‐I). Taken together, these findings suggest that for AQP1‐expressing GBM cells to enhance the width and area covered by ECV304 endothelial cells in vitro, direct contact between ECV304 cells and GBM cells is required.

### 3.7 GBM cells engineered to express AQP1 express reduced levels of *THSD7A*

**FIGURE 6:**
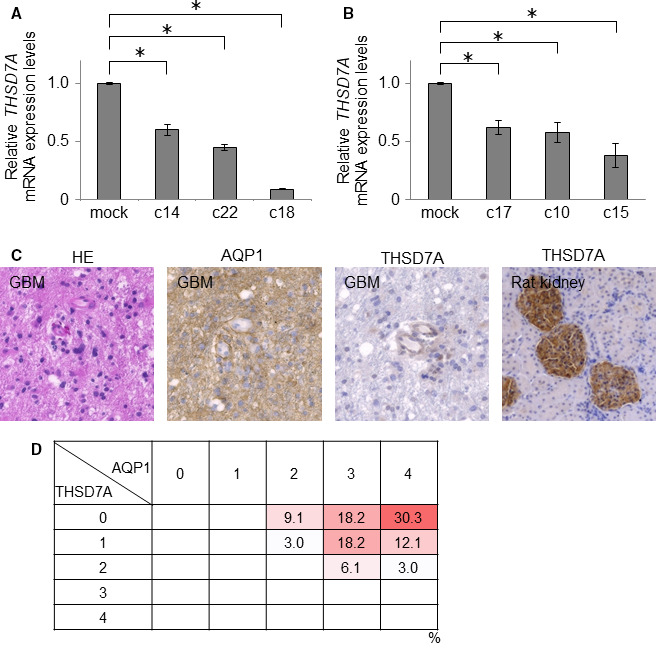
The expression of aquaporin 1 (AQP1) and thrombospondin type 1 domain‐containing 7A (THSD7A) are negatively correlated in human GBM specimens. The comparative expression of *THSD7A* by qRT‐PCR in U251 (A) and U87 clones (B); data represent the mean ± SD, n = 3; *P < .05 for AQP1‐expressing GBM clones vs their mock‐transfection controls. C, Representative images of hematoxylin and eosin (HE) staining (left‐most image) and immunohistochemical analysis of AQP1 and THSD7A (central two images) in a clinical GBM specimen section. Rat kidney was used as the positive control for THSD7A immunostaining (right‐most image). D, Summary of the 33 GBM cases shows a strong negative association between the expression of AQP1 and of THSD7A

To identify the potential molecular mechanisms responsible for the effects of AQP1‐expressing GBM cells on ECV304 endothelial cells cocultured with them in vitro, we isolated total RNA from U251‐c18 cells (the transfectants that express the highest levels of AQP1) and U251 mock‐transfected control cells. We identified differentially expressed genes using data generated with an Affymetrix GeneChip human genome array. We found that 375 genes were upregulated and 430 genes were downregulated in U251‐c18 cells compared with their controls. After identifying the 40 most‐upregulated and 40 most‐downregulated genes (Figure S1), we did a literature search to assess any potential associations of these genes with tumorigenesis. We chose four upregulated genes (*MAGEA3*, *MAGEA6*, *NTM*, and *CNTNAP2*) and five downregulated genes (*ARMCX1*, *SULF1*, *RPRM*, *LBH*, and *THSD7A*) for further validation. We performed qRT‐PCR to confirm AQP1‐dependent expression of these mRNAs. We found that only *THSD7A* was clearly and significantly associated with AQP1 expression in both U251 and U87 cells (Figure 6A,B), and previous studies have demonstrated that THSD7A may regulate angiogenesis.^16^, ^17^

### 3.8 The expression of AQP1 and THSD7A are negatively associated in human GBM specimens

To determine if there is also a negative association between AQP1 and THSD7A expression in the tumors of patients with GBM, we assessed their expression in sections of 33 different GBM clinical specimens by immunohistochemistry (Figure 6C,D). We found that AQP1 was expressed by GBM tumor cells, but these AQP1‐positive tumor cells did not co‐express THSD7A (Figure 6C). Quantitative analyses showed a strongly negative association between the expression levels of AQP1 and THSD7A (Figure 6D). These data indicate that AQP1 downregulates THSD7A expression in GBM tumor cells, and suggests that AQP1 enhances the malignant phenotype of GBM cancer cells and the formation of vascular beds in GBM tumors.

## 4 DISCUSSION

Consistent with prior studies, we found that AQP1 is expressed in GBM tumor cells.^8^, ^12^ AQP1 expression was especially high in the perivascular foci within the boundary of the normal brain and the tumor tissue (Figure 1). This finding is consistent with a previous report,^8^ and with a study that revealed an association between increased AQP1 expression and tumor malignancy in high‐grade astrocytomas.^6^ We thus hypothesized that AQP1 may be involved in cell proliferation, migration, and invasion, as well as in the angiogenesis and the formation of vascular beds in GBM. To explore the function of AQP1 in vitro, we used human U251 and U87 GBM cells. The basal levels of AQP1 expression in U251 and U87 cells were extremely low; therefore, we employed an overexpression technique to investigate the role of AQP1 in these cells (Figure 2). Cell proliferation was not affected by AQP1 expression in either cell line (Figure 3A,B); however, we found that AQP1 expression promoted cell migration and invasion in vitro (Figure 3C‐J). These results are consistent with the findings of a previous study in which AQP1 was overexpressed in human HT20 colon cancer cells.^9^ Similar to our findings, this study found that migration was enhanced in AQP‐1‐expressing melanoma cells.^10^

To support their strong capacity for invasion and angiogenesis, GBM cells express various proteases. Upregulation of cathepsin B, a lysosomal cysteine protease, has previously been reported in the perivascular area, and prior studies have shown that cathepsin B is involved in the migration and invasion of GBM cells, as well as in angiogenesis.^8^, ^22^, ^24^, ^25^, ^26^ Our results showed that the expression of AQP1 induced cathepsin B expression. This may, in turn, contribute to the invasive potential of GBM cells in the perivascular zone, where AQP1 is upregulated. MMP‐9 is known to be involved in cancer initiation, invasion, angiogenesis, and metastasis in a variety of cancer cell types.^27^, ^28^ In lung cancer cells, AQP1 knockdown had inhibitory effects on cancer cell proliferation and migration along with the downregulation of MMP‐9 expression.^28^ It was also demonstrated that silencing of AQP1 expression resulted in decreased MMP‐9 expression in GBM cells.^29^ In this study, forced expression of AQP1 also significantly upregulated MMP‐9 activities (Figure 3M).

Our results also demonstrated that the expression of AQP1 induced the expression of FAK. FAK is a nonreceptor tyrosine kinase that functions within dynamic protein complexes, called focal adhesions, that are present at the site of attachment between cells and the extracellular matrix. FAK can trigger multiple downstream intracellular signaling cascades required for cell survival, growth, adhesion, migration, and invasion.^23^, ^30^, ^31^ In addition, FAK expression is associated with angiogenesis in high‐grade gliomas, and there is evidence that FAK promotes angiogenesis in glioma by activating endothelial cell migration.^32^ Increased FAK expression may also contribute to migration and invasion in the perivascular zone where AQP1 is upregulated.

In this study, we also found morphological changes in the cord‐like structures formed by ECV304 endothelial cells that were grown in direct cocultures with AQP1‐expressing GBM cells. The calculated width of the cord‐like structures was greater in ECV304 cells cocultured with AQP1‐expressing GBM cells than control cells (Figure 4), whereas the length of cord‐like structures was unaffected. GBM tumors are highly vascularized with an abundance of disorganized microvessels and an aggressive proliferation of endothelial cells and pericytes. There are five types of microvascular patterns in GBM: microvascular sprouting, vascular clusters (focal aggregations), vascular garland‐like formations, glomeruloid vascular proliferation, and vasculogenic mimicry.^33^, ^34^ Vascular garland‐like formation, glomeruloid vascular proliferation, and vasculogenic mimicry patterns have been associated with poor outcome in GBM patients.^35^

To unveil the molecular mechanisms underlying the morphological changes in cord‐like structures due to AQP1 overexpression in GBM cells, we performed GeneChip analysis for identification of differentially expressed genes. We identified *THSD7A* as the only gene that was significantly affected by AQP1 in the two GBM cell lines used in this study. THSD7A is a type I membrane protein and contains several domains and motifs, including ten thrombospondin‐I repeats (TSRs) that regulate angiogenesis.^16^ THSD7A contains six extracellular WSXW motifs located in the TSRs. The WSXW motif in thrombospondin‐I can bind to and activate latent transforming growth factor (TGF)‐β. It can also interact with fibronectin, regulate fibronectin‐mediated adhesion, and promote cell adhesion by binding to heparin and heparin sulfate. The WSXW motif is also critical in promoting cell spreading and FAK phosphorylation. Wang et al reported that neural Thsd7a mediated endothelial cell migration during angiogenesis and suggested that Thsd7a plays an important role in the growth path orientation of vascular sprouting in zebrafish.^18^ Based on these data, we speculate that the downregulation of THSD7A in GBM tumor cells affects microvascular formation in GBM. This is the first study, to our knowledge, to unveil the relationship between AQP1 and THSD7A. However, it is still unknown how AQP1 expression could downregulate THSD7A level in GBM cells. Further experiments are required to verify our findings of the association among AQP1, THSD7A, and vascular formation.

The possible explanation behind the relationship between AQP1 and THSD7A is summarized in Figure S2. Our findings suggest that the upregulation of AQP1 increases the aggressive cellular phenotypes of GBM cells and might lead to disorganized microvessels in GBM through THSD7A inhibition. Furthermore, we identified THSD7A as a novel tumor‐associated factor in the most malignant type of brain tumor. Therefore, we suggest that AQP1 is a potential molecular target for the treatment of GBM.

## 5 Ethics approval and consent to participate

All procedures used in this research were approved by the Kanazawa University Medical Ethics Committee.

## CONFLICT OF INTEREST

The authors declare no conflicts of interest.

## AUTHORS' CONTRIBUTIONS

MO designed and performed experiments, analyzed data, and wrote the paper. YH designed experiments and analyzed data. SM and AH performed experiments and analyzed data. MN supervised the research. YY supervised the research and cowrote the paper.

## CONSENT FOR PUBLICATION

All patients provided written informed consent. The study was performed in accordance with the Declaration of Helsinki.

## Supporting information

Fig S1

Fig S2

## Data Availability

The datasets used and/or analyzed during the current study are available from the corresponding author on reasonable request.
