# Supplementary figures and images for: Aquaporin 1 elicits cell motility and coordinates vascular bed formation by downregulating thrombospondin type‐1 domain‐containing 7A in glioblastoma
Source: Cancer Med. 2020 Apr 6;9(11):3904–17. doi: 10.1002/cam4.3032 (PMC7286445; doi:10.1002/cam4.3032)

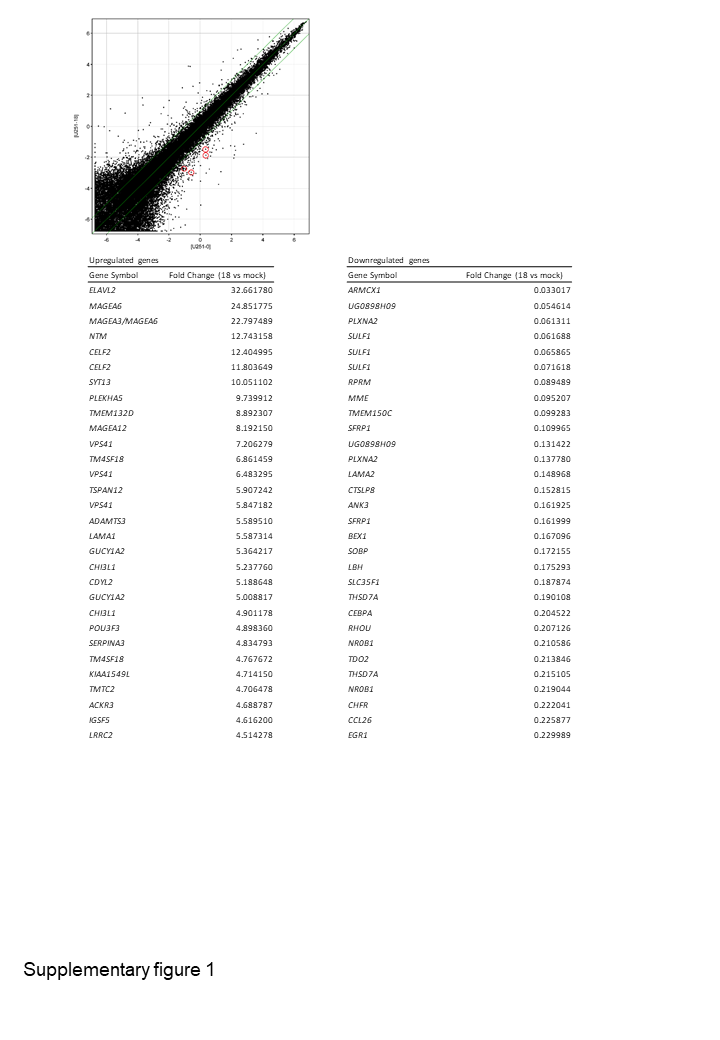

Supplement: Supplementary file 1 — Fig S1 [file CAM4-9-3904-s001.TIF]

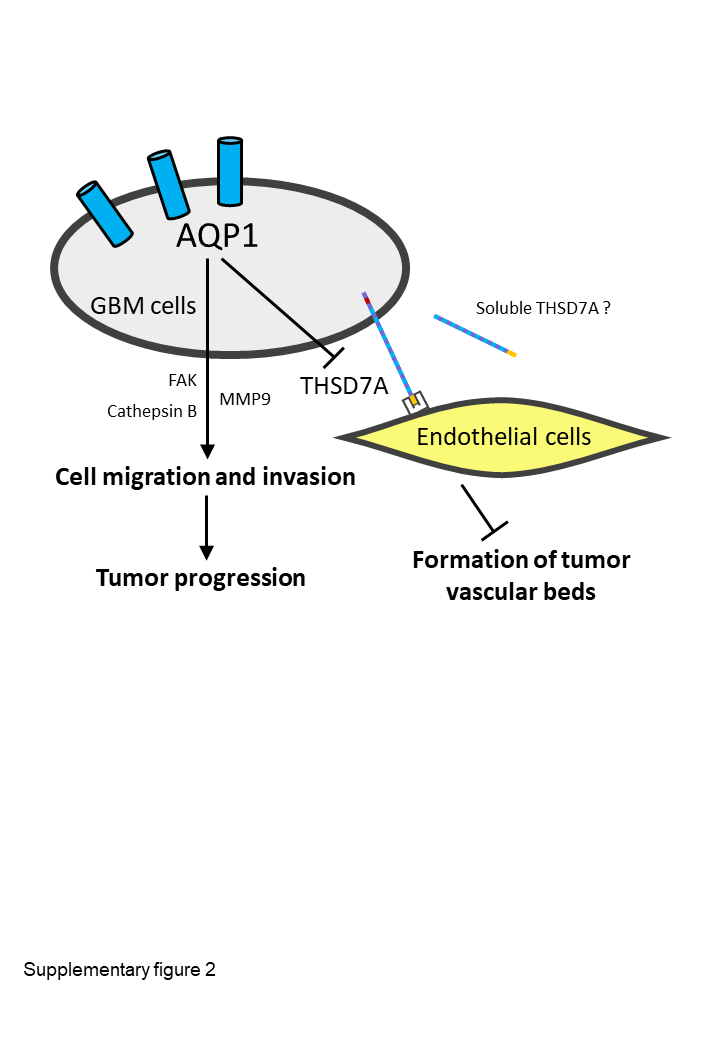

Supplement: Supplementary file 2 — Fig S2 [file CAM4-9-3904-s002.TIF]
